# Analysis and Reporting of Adverse Drug Reactions of Cosmetics at a Tertiary Care Hospital

**DOI:** 10.7759/cureus.56856

**Published:** 2024-03-24

**Authors:** Elisha Paikray, Diptiranjani Bisoyi, Anima Rout, Vedvyas Mishra

**Affiliations:** 1 Pharmacology, Kalinga Institute of Medical Sciences, Kalinga Institute of Industrial Technology (KIIT) University, Bhubaneswar, IND; 2 Skin and Venereal Disease, SCB (Srirama Chandra Bhanja) Medical College and Hospital, Cuttack, IND; 3 Pharmacology, SCB (Srirama Chandra Bhanja) Medical College and Hospital, Cuttack, IND

**Keywords:** product lifestyle management, pharmaco-vigilance, adverse drug reactions, colipa method, cosmeto-vigilance

## Abstract

Background and objectives

Cosmetovigilance is a term used for the activities related to the collection, evaluation, and monitoring of spontaneous reports of undesirable events observed during or after normal or reasonably foreseeable use of a cosmetic product. It is now considered a part of the public health system to determine the toxicity of cosmetic products. India has a pharmacovigilance system that monitors adverse drug reactions. Adverse effects due to the use of cosmetic products are undernumbered and a rigorous vigilant system is necessary to check the unmet needs of our country. Hence keeping the above in view, the study was conducted.

Material and methods

Patients of any gender, aged above 18 years, reporting adverse reactions to cosmetics at the study site were included in the study. The adverse reactions to cosmetics were noted. The causality of the observed adverse cosmetic reactions (ACRs) was done by the European Cosmetic, Toiletry and Perfumery Association (COLIPA) and Product Lifecycle Management (PLM) methods.

Results

A total of 120 patients were included in the study. The cosmetic products used by the patients were mainly face care products (n=144) followed by make-up care products (n=126). A total of 121 types of ACRs were reported. The majority of the ACRs were caused by products involved in facial care (62; 51.2%) followed by personal care products (20; 16.5%). In the causality assessment of the ACRs using the COLIPA method, 49.4% of patients were categorized as likely, and using the PLM method, 59% of the events were categorized as probable.

Conclusion

Most of the ACRs were caused by face care products and acne was reported as the most frequently occurring ACR. Awareness programs regarding the reporting of ACRs should be encouraged among cosmetic users and stakeholders.

## Introduction

In the India Drugs and Cosmetics Act 1940 and Rules 1945 which regulates the Cosmetics Drugs and Cosmetics Act, Section 3 (aaa) defines cosmetics as "articles intended to be rubbed, poured, sprinkled, or sprayed on, or introduced into, or otherwise applied to, the human body or any part thereof for cleansing, beautifying, promoting attractiveness, or altering the appearance, and include any article intended for use as a component in cosmetics" [1]. According to the United States Food and Drug Administration (FDA), cosmetics are defined as “articles for beautification, cleansing, or altering the physical appearance” [2].

The use of cosmetics can help consumers enhance their beauty but they are associated with various adverse reactions. According to the World Health Organization (WHO), an adverse drug reaction is defined as an unintended and noxious response to a cosmetic that normally occurs after a correct application of a cosmetic, whereas an adverse cosmetic event (ACE) is an anticipated noxious injury hypothetically related to cosmetic use [3-5]. While preparing a cosmetic product, it is very vital to maintain the stability of the product because if there is any change in temperature or humidity, it can lead to degradation of the cosmetic ingredients leading to more adverse effects.

"Cosmetovigilance” is a term used for the activities related to the collection, evaluation, and monitoring of spontaneous reports of undesirable events observed during or after normal or reasonably foreseeable use of a cosmetic product [6]. Vigan was the first person to coin the term cosmetovigilance. It refers to the post-marketing surveillance conducted by the industry [7]. The French health products safety agency initiated cosmetovigilance in association with pharmacovigilance for monitoring the adverse effects of the use of cosmetics. It is now considered a part of the public health system to determine the toxicity of cosmetic products [8].

A major proportion of consumers of cosmetic products are more focused on the short-term consequences on appearance and do not think about the long-term consequences on the whole body. Cosmetic products available in the commercial market are regarded to be safe and tolerable [9]. Nowadays there is more thrust on the testing of cosmetic products to determine the harmful effects of cosmetics. Numerous studies have demonstrated that the various chemicals found in commercially accessible cosmetic products pose a health risk to users [10]. The adverse effects can range from a minor hypersensitive reaction to a severe, potentially fatal anaphylactic reaction. The side effects can appear soon after using the cosmetic goods or can take a long time to manifest [10]. Headache, vertigo, fatigue, and nausea are some of the most often seen negative effects of continuous usage of cosmetic items [11].

The main purpose of cosmetovigilance is the collection and assessment of the untoward side effects occurring in people using cosmetics so that the adverse reactions can be reported in a proper format, routine laboratory tests for quality control can be done and lastly, public health concerns can be addressed.

India has a pharmacovigilance system that monitors only adverse drug reactions. The number of adverse reactions reported due to the use of cosmetic products is comparatively low because of self-diagnosis (due to the absence of medical consultation) and self-medication, which are common in the presence of mild to moderate reactions involving the skin. Nevertheless, adverse effects due to the use of cosmetic products are undernumbered even in the presence of medical consultation, so a rigorous vigilant system is necessary to check the unmet needs of our country. Hence keeping the above in view, the study was conducted.

This study aimed to study the spectrum of adverse cosmetic reactions and to assess the causality, predictors, and utilization pattern of ACEs.

## Materials and methods

This was a one-year study conducted at the Department of Pharmacology of SCB Medical College & Hospital, Cuttack, Odisha, India, in collaboration with the Department of Skin and Venereal Disease. Baseline information such as demographic details of the patients, type of cosmetics used, cosmetic utilization pattern, and ADRs were collected by the principal investigator from the outpatient department and were documented in a pre-designed case collection form. Patients of any gender, aged above 18 years, reporting adverse reactions to cosmetics at the study site were included in the study. The study excluded patients who underwent irreversible cosmetic procedures such as plastic surgery, tattoos, fillers, and botox. The Institutional Ethics Committee of SCB Medical College & Hospital approved the study (approval number: 1139, dated October 29, 2022). Each patient provided a written agreement to participate in the study.

According to earlier literature, the percentage of adverse effects attributable to using different forms of cosmetics ranged from 8% to 38% [12]. The sample size was approximated to 120 using a confidence limit of 95% and a margin of error of 5%. The causality of the observed ADR was done by the European Cosmetic, Toiletry and Perfumery Association (COLIPA) and Product Lifecycle Management (PLM) methods [13,14]. The questionnaire for data collection was adopted and modified from previous studies for cosmetic utilization behavior and adverse reactions [15].

Statistical analysis

The data analysis was done using IBM SPSS Statistics for Windows, Version 24.0 (Released 2016; IBM Corp., Armonk, New York, United States). Descriptive statistics were used for analyzing the sociodemographic adverse events, spectrum of adverse events, and causality assessment of adverse cosmetic reactions (ACRs). To determine the strength of association and various predictors of ACRs, 95% confidence interval (CI) and odds ratio have been used, and a p-value <0.05 was considered statistically significant.

## Results

Patients included in the study belonged to both urban as well as rural populations. Females outnumbered the male patients. Fifty-eight patients (48.3%) belonged to the age group of 18-29 years. A major proportion of the study population had passed the class 10 exam. The majority of patients were married. Twenty-five patients (20.9%) and 23 patients (19.1%) had allergies to food and medicine, respectively. Forty-eight patients (40%) had a family history of allergy. Table 1 gives the sociodemographic details of the subjects.

**Table 1:** Sociodemographic details of study participants The data is presented as number (n) and percentage (%).

Characteristics		Number (n)	Percentage (%)
Gender	Male	25	20.8
	Females	95	79.2
Age	18-29 years	58	48.3
	30-40 years	43	35.8
	>41	19	15.8
Marital status	Married	81	67.5
	Unmarried	39	32.5
Education	Literate	76	63.3
	Illiterate	44	36.7
Allergy to food	Yes	25	20.9
	No	95	79.1
Allergy to any medicine	Yes	23	19.1
	No	97	80.9
Family history of allergy	Yes	48	40
	No	72	60

The types of cosmetics used by the patients were categorized into five classes. They were products used for personal care, perfumes and deodorants, nail care, face care, and makeup, which were further sub-classified. Face care products (n=144) followed by make-up care products (n=126) were the most used products. The total number of ACRs reported was 121. The majority of the ACRs were caused by products involved in facial care (n=62; 51.2%) followed by personal care products (n=20; 16.5%). Makeup, hair care, and nail care products accounted for 20 (16.5%), 11 (9%), and three (2.4%) events, respectively.

**Figure 1:**
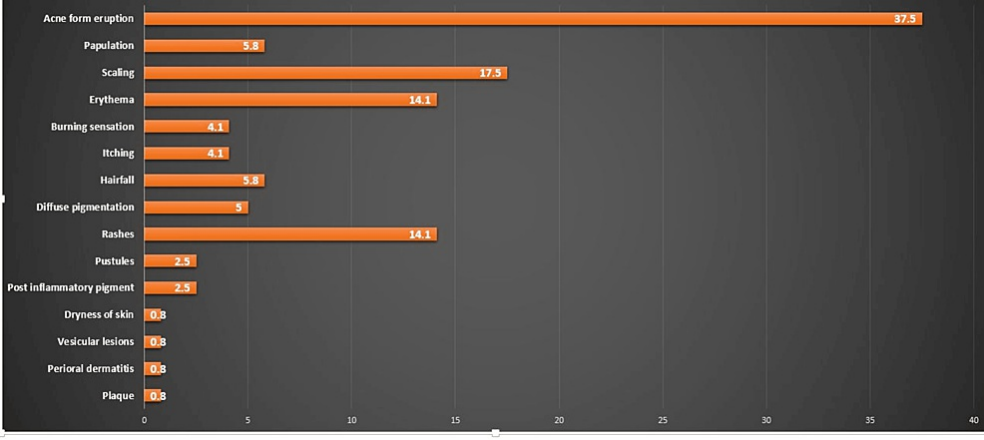
Spectrum of adverse cosmetic reactions The data is presented as percentages

A total of 15 types of ACRs were reported. The analysis of the spectrum of ACRs is shown in Figure 1. The most common ACR was acne-form eruption. This was followed by scaling, erythema, and rashes. Other types of adverse events were burning sensation, itching, hair fall, pigmentation, post-inflammatory pigment, dryness of skin, vesicular lesions, perioral dermatitis, and plaque.

**Figure 2:**
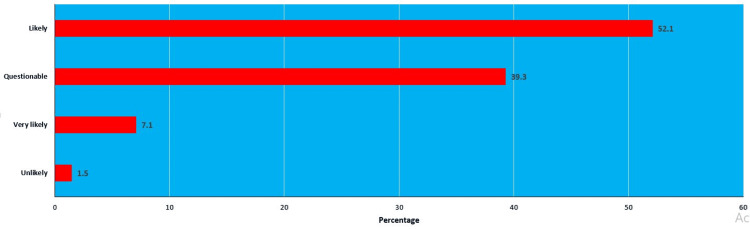
Causality assessment using COLIPA method Data is presented as percentages. COLIPA: European Cosmetic, Toiletry, and Perfumery Association

The causality assessment of the adverse events during cosmetics use as per the COLIPA method was categorized into likely, questionable, very likely, and unlikely. The number of patients categorized into likely was 63 (52.1%) whereas questionable, very likely, and unlikely were 48 (39.3%), eight (7.1 %), and two (1.5 %), respectively (Figure 2).

**Figure 3:**
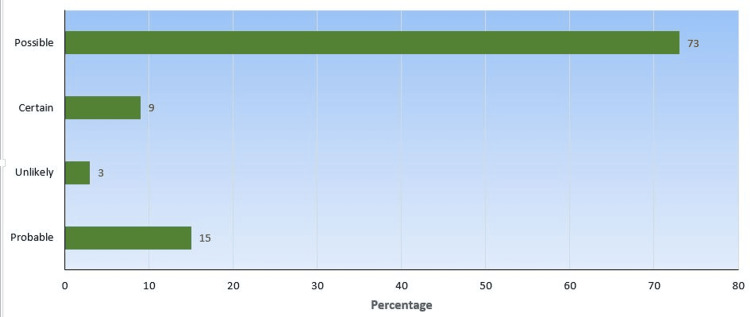
Causality assessment using PLM method Data is presented as percentages PLM: Product Lifecycle Management

As per the causality assessment by the PLM method, 88 (73%) events were categorized as possible, 11 (9 %) as certain, 18 (15%) as probable, and four (3%) as unlikely, as shown in Figure 3.

The statistical analysis showed that parameters like gender, age group, number of cosmetics used, duration of cosmetics use, sharing of cosmetics, history of allergy to food, cosmetics, and family history of allergy to cosmetics were statistically insignificant, as shown in Table 2.

**Table 2:** Predictors of adverse cosmetic reactions P-value less than 0.05 is considered to be significant.

Variables		Odds Ratio	95% CI	p-value
Gender	Male	0.225	0.028-1.802	0.1
	Female	-	-	
Age (In years)	18-29	0.62	0.193-2.008	0.4
	30-40	-	-	0.4
	>41	1.63	0.186-2.3	0.5
Number of cosmetics used	<2	1.765	0.472-6.856	0.1
	2-5	0.821	0.233-2.887	0.3
	>5	0.608	0.064-5.324	0.6
Duration of cosmetics use	<6 months	3.409	0.652-17.822	0.13
	6 months-1 year	3.000	0.459-19.593	0.2
	>2 years	0.992	0.285-3.458	0.9
Allergy to food	Yes	0.625	0.122-3.198	0.5
Allergy to any medicine	Yes	1.043	0.364-3.467	0.9
Family history of allergy	Yes	--	0.274-3.642	0.9
Sharing of other cosmetics	Yes	1.016	0.261-3.949	0.9

## Discussion

Cosmetovigilance is a new concept under pharmacovigilance. This study gives a clear analysis of ACRs and cosmetics causing it. The results found in this study should be compared against the findings of similar studies conducted at different places to further strengthen the concept of cosmetovigilance. This study comprises 120 study participants. Females accounted for a higher proportion of ACRs, which was similar to the studies by Bilal et al. [14] and Di Giovanni et al. [4]. The majority of the patients (n=58; 48.3 %) belonged to the age group of 18-29 years. This finding was similar to the study conducted by Bilal et al. [14] and Norudi et al. [15]. Most of the study population was literate and married.

Facial care products (n=144; 62%) followed by personal care (n=50; 21.4%) and make-up products (n=38; 16.5%) were the commonest culprits for causing ACRs. This finding is similar to the findings of Sportiello et al. [16]. However, this is in contrast to the findings of Getachew and Twelde [17] and Meharie et al. [18], which reported hair dyes were responsible for maximum ACRs. Patch test was not performed to identify the causative agents but certain substances like stearic acid, Ceteareth-20, PEG-40 castor oil, and PEG-100 stearate can cause inflammation and various adverse events, particularly at higher concentrations [19-22]. Naturally derived products like aloe vera, balsam, and cucumber can also cause adverse cosmetic reactions [23,24]. Factors such as seasonal variations, hormonal imbalance, bacterial, parasite, and fungal infection, and psychological problems such as stress and unhygienic practices can be responsible for acne-form eruptions. 

Cosmetic products cause an array of adverse reactions. A total of 15 types of ACRs were reported in this study from different patients. The total number of ACRs reported was 121. The most frequent ACR was acne-form eruption (n=45; 37.5%) followed by scaling (n=21; 17.5%), and erythema and rashes (n=17; 14.1% each). This is in line with the findings of Getachew and Twelde [17]. Other types of ACRs were papulation, hair fall, diffuse pigmentation, itching, rashes, pustules, post-inflammatory pigment, dryness of skin, vesicular lesions, perioral dermatitis, and plaque. The reported adverse events in a study conducted in Ethiopia were allergic reactions (36%), acne (16%), and skin thinning (9.6%) [14]. Another study reported events such as itching (68.9%), redness (61%), acne (14.2%), and scaling of hair (19.3%) by frequent use of products classified as cosmetics [25]. Factors such as seasonal variations, hormonal imbalance, bacterial, parasite, and fungal infection, and psychological issues such as stress and unhygienic practices can be responsible for ACRs [26]. 

COLIPA method was used for causality assessment and the results are as follows: 63 were likely (52.1%) whereas 48 (39.3%) were questionable, eight (7.1%) were very likely, and two (1.5%) were unlikely. In another study, the ACRs were categorized as very likely in 30% of events, likely in 18%, unlikely in 16%, questionable in 18%, and unknown in 18% [26]. The PLM method categorized 88 (73%) events as possible, 11 (9%) as certain, 18 (15%) as probable, and four (3%) as unlikely, which was mostly similar to a previous study [27].

The study had some limitations. The patient population was collected from the dermatology department only whereas patients from other departments too could have been affected. The subsequent management of ACRs was not noted and their pharmacoeconomic analysis was not done.

## Conclusions

The safety of cosmetics is monitored through a novel idea called cosmetovigilance. The majority of patients in the current study reported having at least one one adverse reaction. Most of the ACRs were caused by face care products and acne was reported as the most common ACR. Awareness programs regarding the reporting of ACRs should be encouraged among cosmetic users and stakeholders.
